# The V86M mutation in HIV-1 capsid confers resistance to TRIM5α by abrogation of cyclophilin A-dependent restriction and enhancement of viral nuclear import

**DOI:** 10.1186/1742-4690-10-25

**Published:** 2013-02-28

**Authors:** Maxime Veillette, Katsiaryna Bichel, Paulina Pawlica, Stefan M V Freund, Mélodie B Plourde, Quang Toan Pham, Carlos Reyes-Moreno, Leo C James, Lionel Berthoux

**Affiliations:** 1 Department of Medical Biology, Laboratory of retrovirology and GROEM, Université du Québec à Trois-Rivières, 3351 Boulevard des Forges, CP500, Trois-Rivières, QC G9A 5H7, Canada; 2 Medical Research Council Laboratory of Molecular Biology, Division of Protein and Nucleic Acid Chemistry, Hills Road, Cambridge CB2 0QH, United Kingdom

## Abstract

**Background:**

HIV-1 is inhibited early after entry into cells expressing some simian orthologues of the tripartite motif protein family member TRIM5α. Mutants of the human orthologue (TRIM5α_hu_) can also provide protection against HIV-1. The host protein cyclophilin A (CypA) binds incoming HIV-1 capsid (CA) proteins and enhances early stages of HIV-1 replication by unknown mechanisms. On the other hand, the CA-CypA interaction is known to increase HIV-1 susceptibility to restriction by TRIM5α. Previously, the mutation V86M in the CypA-binding loop of HIV-1 CA was found to be selected upon serial passaging of HIV-1 in cells expressing Rhesus macaque TRIM5α (TRIM5α_rh_). The objectives of this study were (i) to analyze whether V86M CA allows HIV-1 to escape mutants of TRIM5α_hu_, and (ii) to characterize the role of CypA in the resistance to TRIM5α conferred by V86M.

**Results:**

We find that in single-cycle HIV-1 vector transduction experiments, V86M confers partial resistance against R332G-R335G TRIM5α_hu_ and other TRIM5α_hu_ variable 1 region mutants previously isolated in mutagenic screens. However, V86M HIV-1 does not seem to be resistant to R332G-R335G TRIM5α_hu_ in a spreading infection context. Strikingly, restriction of V86M HIV-1 vectors by TRIM5α_hu_ mutants is mostly insensitive to the presence of CypA in infected cells. NMR experiments reveal that V86M alters CypA interactions with, and isomerisation of CA. On the other hand, V86M does not affect the CypA-mediated enhancement of HIV-1 replication in permissive human cells. Finally, qPCR experiments show that V86M increases HIV-1 transport to the nucleus of cells expressing restrictive TRIM5α.

**Conclusions:**

Our study shows that V86M de-couples the two functions associated with CA-CypA binding, i.e. the enhancement of restriction by TRIM5α and the enhancement of HIV-1 replication in permissive human cells. V86M enhances the early stages of HIV-1 replication in restrictive cells by improving nuclear import. In summary, our data suggest that HIV-1 escapes restriction by TRIM5α through the selective disruption of CypA-dependent, TRIM5α-mediated inhibition of nuclear import. However, V86M does not seem to relieve restriction of a spreading HIV-1 infection by TRIM5α_hu_ mutants, underscoring context-specific restriction mechanisms.

## Background

TRIM5α was isolated as a retroviral restriction factor in 2004 [1] and acts within the post-entry, pre-integration window [2,3]. The viral molecular target of TRIM5α is the correctly matured N-terminal domain of capsid (CA) proteins forming the outer surface of the retroviral core [2,4-8]. A direct interaction between the two proteins, each present as high molecular weight multimers, occurs shortly after entry and is required for downstream inhibition of viral replication [8-12]. The mechanism of TRIM5α-mediated restriction can be broken down to discrete events, some of them inter-dependent: (i) virus entrapment into TRIM5α cytoplasmic bodies [13], (ii) decreased stability of the virus core [8,14-16], (iii) targeting to a proteasome-dependent degradation pathway [17-20], and (iv) inhibition of nuclear transport [17,21,22].

CypA, a host peptidyl-prolyl *cis*/*trans* isomerase that is ubiquitously expressed in tissues, is known to play roles in both HIV-1 infection of human cells and in HIV-1 restriction by TRIM5α in monkey cells. In dividing permissive human cells, CypA enhances HIV-1 infectivity by regulating the disassembly of its core [23-25] independently of TRIM5α [26,27]. On the other hand, restriction of HIV-1 by simian TRIM5α orthologues is enhanced by CypA, and inhibition of CypA expression or of its activity partially rescues infectivity in restrictive conditions [21,26,28-30]. CypA binds to an exposed proline-rich loop on the viral CA [31,32] and catalyzes the isomerisation of the peptide bond G89-P90 [33,34]. The mutation V86M in the CypA-binding loop of HIV-1 CA has been identified as conferring partial resistance to TRIM5α_rh_[35]. The mechanism of HIV-1 resistance to TRIM5α_rh_ conferred by V86M CA was not addressed in this study. However, it was established that this mutation in the CypA-binding loop did not disrupt CA-CypA interactions *in vitro* or in cell cultures [35].

We and others have proposed that point mutations in the variable region 1 (v1) of TRIM5α_hu_ could confer HIV-1 restriction capability [36-41]. These mutations were discovered by mapping of HIV-1 restriction determinants in non-human TRIM5α orthologues [36,39-41] or through the use of random mutagenesis-based screens [38,42]. Such antiviral genes are promising candidates for gene therapy applications, owing to a few key characteristics: (i) They block replication early after virus entry and before integration can occur; (ii) They are human-like and thus probably nonimmunogenic; (iii) They inhibit HIV-1 by a well-established, natural mechanism with little side effect expected. However, it is currently unknown whether HIV-1 can acquire resistance to TRIM5α_hu_ mutants. Here we investigate the extent and mechanism of resistance of the HIV-1 CA mutant V86M to R332G-R335G TRIM5α_hu_ and other mutants of the v1 domain. Our data show that this mutation affects physical and functional interactions with CypA in order to decrease HIV-1 sensitivity to TRIM5α while retaining replication-enhancement functions also conferred by CypA binding.

## Results

### CA-V86M HIV-1 is partially resistant to restriction by TRIM5α_hu_ mutant R332G-R335G

**Figure 1:**
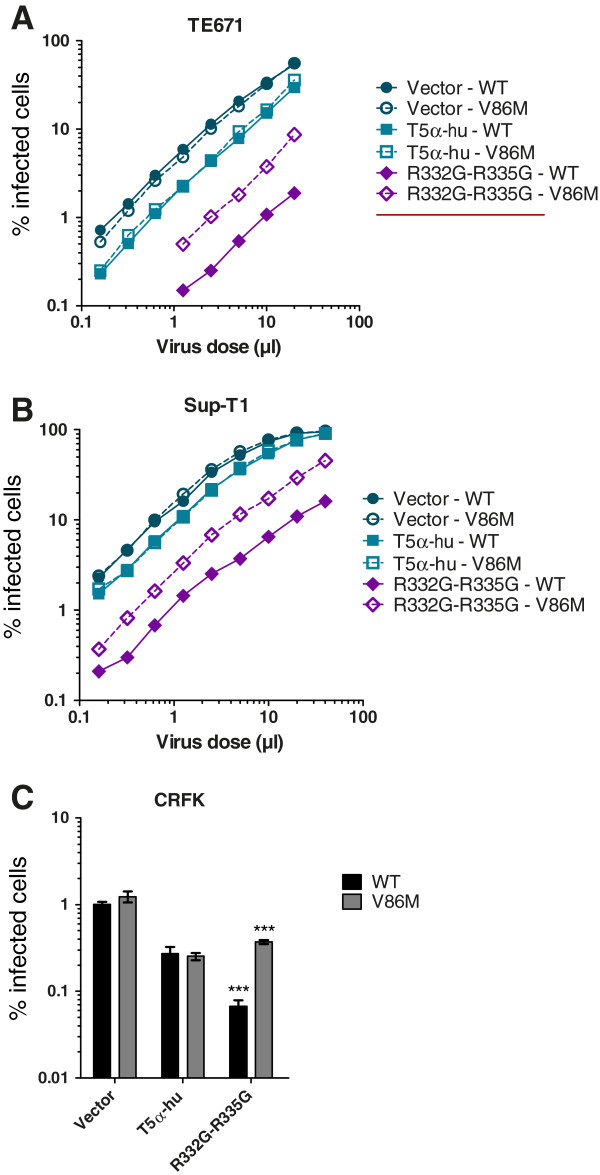
**V86M mutation in HIV-1 capsid confers partial resistance against R332G-R335G TRIM5α**_**hu**_**. **TE671 (**A**), Sup-T1 (**B**) and CRFK (**C**) cells were transduced with either WT TRIM5α_hu_, R332G-R335G TRIM5α_hu_, or with the “empty” vector as a control. Following antibiotic selection, cells were infected with multiple doses (**A**, **B**) or with a single dose (**C**) of WT or V86M HIV-1_NL-GFP_. The two viral preparations had similar virion content as determined by RT assay. The percentage of infected (GFP-positive) cells was determined two days later by flow cytometry. In (**C**), infections were done at a multiplicity of infection of ~0.1 as determined for the WT virus in parental cells. Bars show the average data from 3 infections with standard deviations (***, P-value < 0.0001).

We analyzed whether V86M could protect HIV-1 against restriction by TRIM5α_hu_ mutant R332G-R335G in human TE671, human Sup-T1 and feline CRFK cells. The choice of human cells as an experimental model was justified by the need to gather data in cells representative of the HIV-1 natural host, while the analysis in cat cells was prompted by the absence of an endogenous TRIM5α protein in this species, thus allowing us to analyze restriction in the absence of potentially interfering effects from the endogenous gene. All the cell lines created were challenged by wild-type (WT) or V86M HIV-1 NL43-based vectors (Figure 1). The two vectors had almost identical titers in non-restrictive human or cat cells. In addition, when those vector preparations were normalized according to reverse transcriptase activity or titer in CRFK cells, they were found to have similar titers in activated human lymphocytes or in human M0, M1 and M2 macrophages (Additional file 1: Figure S1). However, V86M HIV-1_NL-GFP_ was about 4-times more infectious than its WT counterpart in cells expressing R332G-R335G TRIM5α_hu_, and this was true in all the three cell lines tested (Figure 1). In other words, restriction of V86M HIV-1_NL-GFP_ was 4-times less efficient than restriction of WT HIV-1_NL-GFP_.

### Restriction of V86M CA HIV-1 by R332G-R335G TRIM5α_hu_ is independent of cyclophilin A

**Figure 2:**
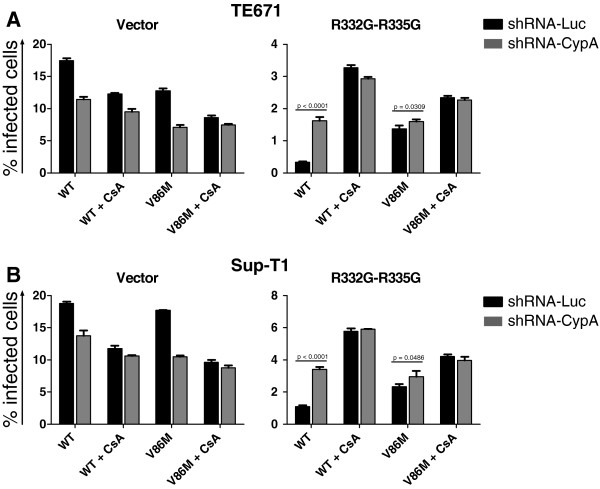
**Restriction of CA-V86M HIV-1 by R332G-R335G TRIM5α**_**hu **_**is independent of cyclophilin A.** TE671 cells (**A**) and Sup-T1 cells (**B**) transduced with either WT TRIM5α_hu_, R332G-R335G TRIM5α_hu_ or with the “empty” vector were also transduced with shRNAs targeting CypA or the non-relevant luciferase mRNAs. Following antibiotic selection, cells were challenged with a single dose of WT or V86M HIV-1_NL-GFP_, in the presence or absence of 2 μM CsA. A multiplicity of infection of ~0.2 was used, as determined for the WT virus in parental cells and in the absence of drug. WT and V86M virus preparations were adjusted by RT assay. Cells were analyzed by FACS two days after infection, and the bars show the average % infected cells from 3 infections with standard deviations. Note the difference in scale between the y-axis of different graphs.

Although V86 is located in the CypA-binding loop of HIV-1 CA, this mutant was previously shown to retain CA binding to CypA [35]. Accordingly, transduction of lymphocytes and macrophages by V86M HIV-1 was significantly affected by treatment by 5 μM of cyclosporine A (CsA), a drug that competes with CA for binding to CypA (Additional file 1: Figure S1). We nonetheless hypothesized that the mutation might affect functional interactions between CypA, TRIM5α and CA. To directly analyze the role of CypA in V86M-mediated resistance, we knocked down its expression in TE671 and in Sup-T1 cells that had been transduced with WT or R332G-R335G TRIM5α_hu_ or with the empty vector. The knockdown was efficient for all the TRIM5 variants and in both cell contexts (Additional file 1: Figure S2). Non-transduced cells were eliminated by antibiotic treatment, and cells were then challenged with either WT or V86M NL4-3 vectors in the presence or absence of 2 μM CsA (Figure 2). In control permissive cells, replication of both WT and V86M HIV-1_NL-GFP_ was decreased (25 to 35%) either by expression of the CypA shRNA or by CsA treatment [25,43] (Figures 2A and 2B, left panels). As expected, combining CsA treatment and CypA knockdown had no additional inhibitory effect on HIV-1 replication in the two cell lines, confirming that CsA inhibits incoming HIV-1 in human cells by interfering with its interactions with CypA [25,27,43]. In TE671 and Sup-T1 cells expressing R332G-R335G TRIM5α_hu_, replication of WT HIV-1 was strongly reduced compared with the control “vector” cells (~50-fold and 20-fold, respectively) and like before, V86M partly rescued infection of HIV-1 in these cells (5-fold in TE671 cells, 2.5-fold in Sup-T1 cells). CypA knockdown enhanced infectivity of WT HIV-1_NL-GFP_ by 5-fold in TE671 cells and by 3-fold in Sup-T1 cells, but had no significant effect on the replication of the V86M mutant. Likewise, CsA treatment strongly increased replication of WT HIV-1_NL-GFP_ in R332G-R335G TRIM5α_hu_ cells (about 10-times in TE671, 5-times in Sup-T1 cells) but had a much smaller effect on V86M HIV-1_NL-GFP_ (1.5- to 2-fold in both cell lines). Therefore, replication of V86M CA HIV-1 in cells expressing R332G-R335G TRIM5α_hu_ was not efficiently rescued by depletion or inhibition of CypA.

### CsA concentration-dependent assays

**Figure 3:**
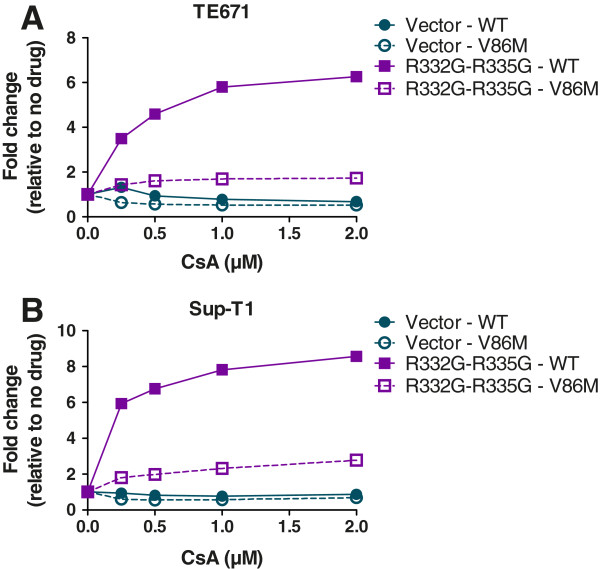
**CsA dose-dependent analysis. **TE671 cells (**A**) and Sup-T1 cells (**B**) expressing R332G-R335G TRIM5α_hu_ or transduced with the “empty” vector were challenged with WT or V86M HIV-1_NL-GFP_ in the presence of increasing concentrations of CsA. Virus doses were adjusted so that approximately 1% of cells were infected in the absence of CsA. The percentages of infected cells were determined two days later as before and results are shown as –fold increases relative to the relevant no-drug controls.

In this experimental setting, instead of using a single volume of HIV-1_NL-GFP_ for all infections as in Figure 2, we adjusted the amount of virus used for each virus/cell combination so that approximately 1% of cells were infected in the absence of drug, and we then repeated the infection in presence of increasing CsA concentrations (Figure 3). Like before, CsA decreased replication of both WT and V86M HIV-1_NL-GFP_ in the permissive control “vector” cells by 30% to 50% in both TE671 cells (Figure 3A) and Sup-T1 cells (Figure 3B). CsA rescued replication of WT HIV-1_NL-GFP_ in cells expressing R332G-R335G TRIM5α_hu_, by up to 6-fold in TE671 cells and 8-fold in Sup-T1 cells. In contrast, the impact of the drug on the restriction of the V86M mutant by this variant of TRIM5α_hu_ was much smaller: up to 1.5-fold and 2-fold in TE671 and Sup-T1 cells, respectively.

Altogether, data in Figures 2 and 3 show that restriction of V86M HIV-1 replication by R332G-R335G TRIM5α_hu_ is poorly sensitive to the presence of CypA compared to restriction of the WT control. However, replication of V86M HIV-1 in permissive human cells is as much sensitive as WT HIV-1 to the presence of CypA.

### The sensitivity of HIV-1 restriction by TRIM5α_hu_ mutants to the V86M mutation correlates with its sensitivity to CsA treatment

**Figure 4:**
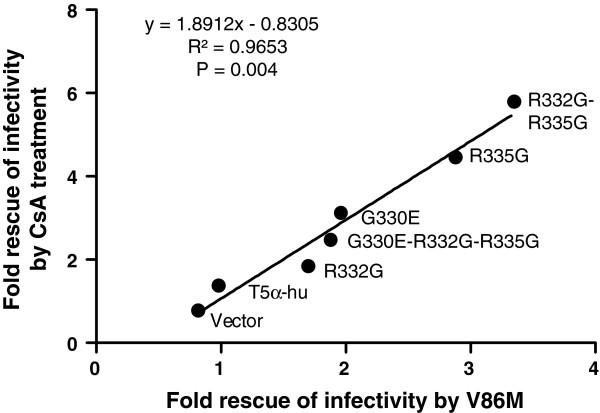
**Direct correlation between the effect of mutation V86M and the effect of CsA treatment. **The increase in HIV-1 infectivity in TE671 cells expressing the indicated TRIM5α_hu_ in presence of 2 μM CsA was plotted against the increase in infectivity resulting from the V86M mutation. All values are –fold increases relative to the untreated WT control virus. Data were gathered at a multiplicity of infection yielding close to 1% infected cells for the untreated WT HIV-1 in the cell lines expressing each the various TRIM5α. The correlation is significant, as shown by the R^2^ obtained and the Spearman’s P-value. The linear regression equation is shown.

Data shown in Figures 1 and 2 revealed that restriction of HIV-1 by R332G-R335G TRIM5α_hu_ was counteracted either by the V86M mutation or by CsA treatment, and suggested that the two interventions had redundant effects. In order to better characterize the role of CypA in the TRIM5α resistance conferred by V86M, we analyzed the effect of this mutation on other mutants of TRIM5α_hu_. We also analyzed the effect of CsA treatment on restriction by these same mutants. G330E, R332G, R335G, and G330E-R332G-R335G TRIM5α_hu_ all restricted HIV-1 when expressed in TE671 cells, albeit with various efficacies (Additional file 1: Figure S3A), and as shown before [38,42]. Replication of HIV-1_NL-GFP_ in these cells was enhanced either by introducing the V86M mutation (Additional file 1: Figure S3A) or by CsA treatment (Additional file 1: Figure S3B). When we plotted the increase in HIV-1_NL-GFP_ infectivity resulting from V86M against the increase in infectivity resulting from CsA treatment in TE671 expressing these various TRIM5α_hu_, we observed a highly significant correlation (Figure 4). Also, the level of rescue of HIV-1_NL-GFP_ infectivity by either V86M mutation or CsA treatment was grossly proportional to the level of restriction. This analysis supports our findings that V86M and CsA treatment rescue HIV-1 replication in restrictive conditions by redundant mechanisms. However, the slope of the linear curve (1.89) also shows that CsA is more efficient than V86M at rescuing HIV-1 replication.

### Restriction of V86M CA HIV-1 by R332G-R335G TRIM5α_hu_ in cat cells is insensitive to CsA

**Figure 5:**
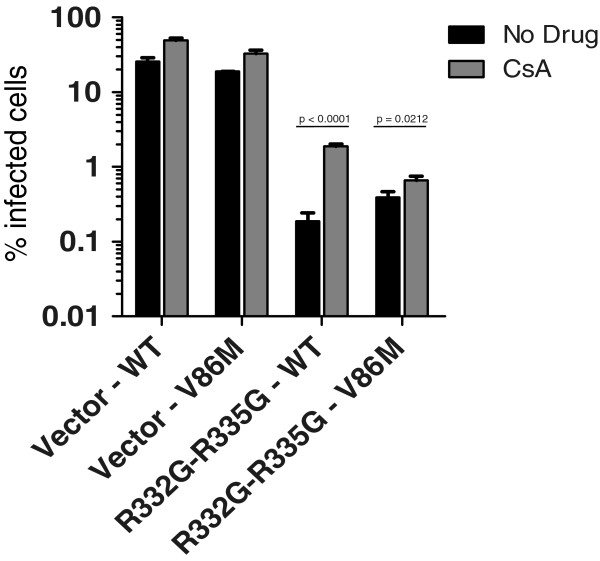
**V86M HIV-1 restriction by R332G-R335G TRIM5α**_**hu **_**in cat cells is not counteracted by CsA treatment. **CRFK cells were transduced with R332G-R335G TRIM5α_hu_ or with the empty vector as control and subsequently challenged with a single dose of WT or V86M HIV-1_NL-GFP_ in presence or absence of 2 μM CsA. Infections were done at a multiplicity of infection of ~0.2 as determined in parental cells infected with the WT virus, and the amounts of viruses used were normalized by RT assay. The percentages of infected cells were determined two days later by FACS.

In human cells expressing an HIV-1-restrictive TRIM5α, CsA treatment might cause dual, opposite effects on HIV-1: enhancement by counteracting TRIM5α, and inhibition by a yet unclear, CypA-dependent mechanism. In addition, although endogenous TRIM5α_hu_ is thought to be recessive against the over-expressed TRIM5α in our experimental system, there was still a slight possibility for interference effects. To address these issues, we analyzed the rescue of HIV-1 replication by V86M mutation and/or by CsA treatment under restriction by R332G-R335G TRIM5α_hu_ in CRFK cat fibroblasts. These cells do not express any TRIM5α, and CsA treatment does not inhibit WT HIV-1 infection in cat cells [26,44]. We found that R332G-R335G TRIM5α_hu_ strongly restricted HIV-1_NL-GFP_, and the mutation V86M in CA decreased restriction by about 3-fold (Figure 5). WT HIV-1_NL-GFP_ replication was enhanced 10-times by CsA treatment while the effect of the drug on the replication of V86M HIV-1_NL-GFP_ was much smaller (1.5-fold), thus mirroring the data obtained in human cells.

### V86M CA interactions with CypA studied by NMR

**Figure 6:**
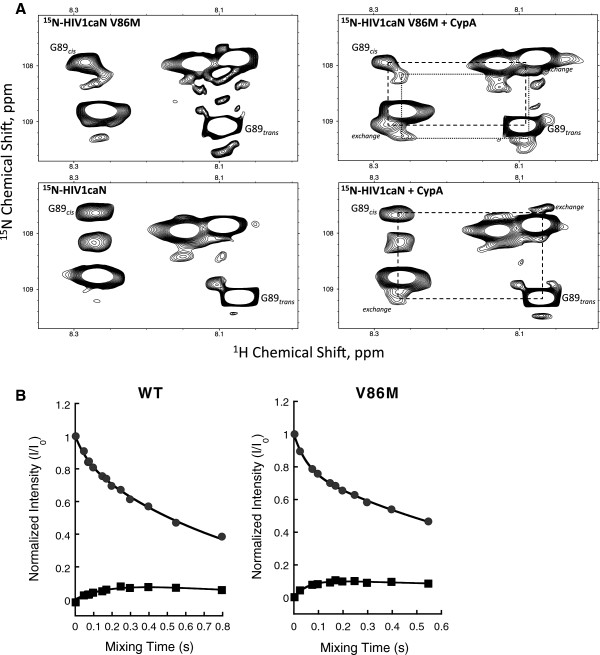
**V86M CA interactions with CypA studied by NMR. **(**A**) Expansions of 2D ^1^H-^15^N ZZ-exchange spectra illustrating CypA catalysed *cis-trans* proline isomerisation of P90 in HIV1caN. The four panels represent backbone amide 1H,15N correlations for the preceding G89 residue in HIV1caN and in HIV1caN V86M in the presence and absence of CypA. In the absence of CypA, the isomerisation reaction is slow and only distinct G89 *cis-* and *trans-* correlation peaks are observed. The presence of CypA leads to an increase in the isomerisation rate and the accumulation of *cis* and *trans* exchange peaks. The dotted box denotes the location of the exchange peaks and indicates their symmetrical distribution with regard to G89_*cis*_ and G89_*trans*_. Note that in the case of HIV1caN V86M a broad G89cis peak and an additional cis-exchange peak indicates a second conformer. All spectra were acquired at the same mixing time (69 ms) and processed with the same contour levels. (**B**) Evaluation of exchange rates for CypA catalyzed isomerisation of G89-P90 in HIV1caN and HIV1caN V86M. Normalised peak intensities of *trans* auto peaks and the corresponding exchange peaks for HIV1caN/CypA and HIV1caN V86M/CypA were plotted as a function of mixing time (s). Exchange rates (k_ex_) in the order of 5 s^-1^ for HIV1caN/CypA and 20 s^-1^ for HIV1caN V86M/CypA were extracted by fitting auto peaks and exchange peaks as described by Bosco et al. (2002).

Data in Figures 1, 2, 3, 4 and 5 showed that V86M made HIV-1 resistant to CypA-dependent, TRIM5α-mediated restriction mechanisms. Thus, we hypothesized that V86M could modify the interactions between HIV-1 CA and CypA, or the isomerisation of the former by the latter [33,45]. The interaction of CA with CypA was studied by 2D ^1^H,^15^N ZZ-exchange NMR spectroscopy (Figure 6). In the absence of CypA, we observed two G89 N-H correlation peaks in the V86M capsid spectra, indicating that, as in WT, the GP bond exists in both *cis* and *trans* states. However, the peak positions for both states in V86M were chemically shifted compared to WT, suggesting that the mutation influences the conformations adopted by the cyclophilin-binding loop (Figure 6A). Furthermore, there is evidence of a second minor G89_*cis*_ peak in the V86M spectra that is indicative of discrete CypA-binding loop isomers.

Upon addition of CypA, symmetrical exchange peaks were observed for WT and V86M indicating that both capsids are a substrate for CypA and that increased *cis-trans* exchange occurs during the ZZ-exchange experiment. However, in the V86M mutant, at least two *cis* exchange peaks were observed compared to a single peak in the WT. Furthermore, the intensities and line shapes of these additional peaks were directly affected by addition of CypA and dependent upon the mixing time of the experiment (Figure 6B). This supports the hypothesis that the V86M mutation results in distinct populations of conformers for the G89-P90 bond.

Next, we determined the catalysed isomerisation rate of the G89-P90 bond in both WT and V86M capsids in the presence of CypA (Figure 6B). For WT capsid we observed a similar rate as previously published, of ~5 s^-1^. For the V86M capsid, the exchange rate for the major G89 peak was ~20 s^-1^. This suggests that the V86M mutation has both altered the conformations adopted by the CypA-binding loop and increased the rate at which it is isomerised. The presence of an additional G89_*cis*_ peak in V86M in the absence of CypA suggests that the mutant capsid may also undergo faster uncatalysed isomerisation, however we were not able to quantify this under the conditions tested.

### Effects of V86M on early stages of viral replication

**Figure 7:**
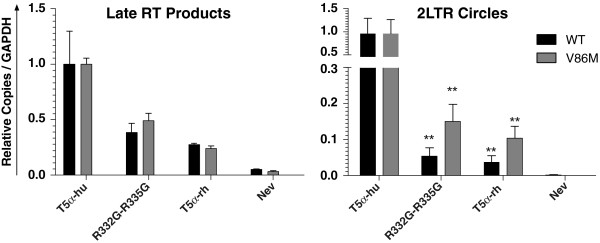
**V86M enhances nuclear transport of HIV-1 DNA in restrictive conditions. **TE671 cells expressing the indicated TRIM5α were challenged with WT or V86M HIV-1_NL-GFP_. An RT inhibitor (nevirapine) control was included to show the absence of contaminating DNA. The levels of HIV-1 late reverse transcription products and 2-LTR circles were determined by real-time quantitative PCR using primers to detect GFP or 2-LTR junctions, respectively. DNA amounts were calculated using serially diluted, linearized DNAs as standards. Data are presented as mean ratio of late RT or 2-LTR DNA copies over the number of GAPDH DNA copies, normalized to the permissive control TRIM5α_hu_. Shown are values with standard deviations of 3 or 5 independent infections (late RT products and 2-LTR, respectively). Student’s *t*-test was used to test for significance of data, specifically WT vs V86M viruses in cells expressing R332G-R335G or TRIM5α (p = 0.0020 and p = 0.0015, respectively).

qPCR assays were performed on DNA extracted from TE671 cells expressing either WT TRIM5α_hu_, R332G-R335G TRIM5α_hu_ or, as a positive control, TRIM5α_rh_, and then infected with WT or V86M HIV-1_NL-GFP_ (Figure 7). We quantified GFP DNA as a marker for late reverse transcription products and 2-LTR circles as a marker for nuclear viral DNA. As expected, accumulation of HIV-1 DNA was reduced in cells expressing TRIM5α_rh_ and R332G-R335G TRIM5α_hu_, and levels of nuclear HIV-1 DNA were even more strongly decreased. This is consistent with HIV-1 replication being impeded by TRIM5α at both the reverse transcription and nuclear transport stages [17,22]. The amounts of viral DNAs were more decreased in TRIM5α_rh_ cells than in R332G-R335G TRIM5α_hu_ cells, consistent with the greater magnitude of restriction conferred by TRIM5α_rh_. V86M had no effect on total DNA levels but significantly increased relative amounts of 2-LTR nuclear DNA species. However, V86M 2-LTR DNA levels in restrictive cells were still reduced 5- to 10-times compared with levels of WT 2-LTR DNA in non-restrictive conditions, as expected from the observation that V86M only partially rescues HIV-1 in restrictive conditions. In summary, escape from restriction by this mutant is associated with increased HIV-1 transport to the nucleus.

### Effect of CypA and V86M on a spreading HIV-1 infection

**Figure 8:**
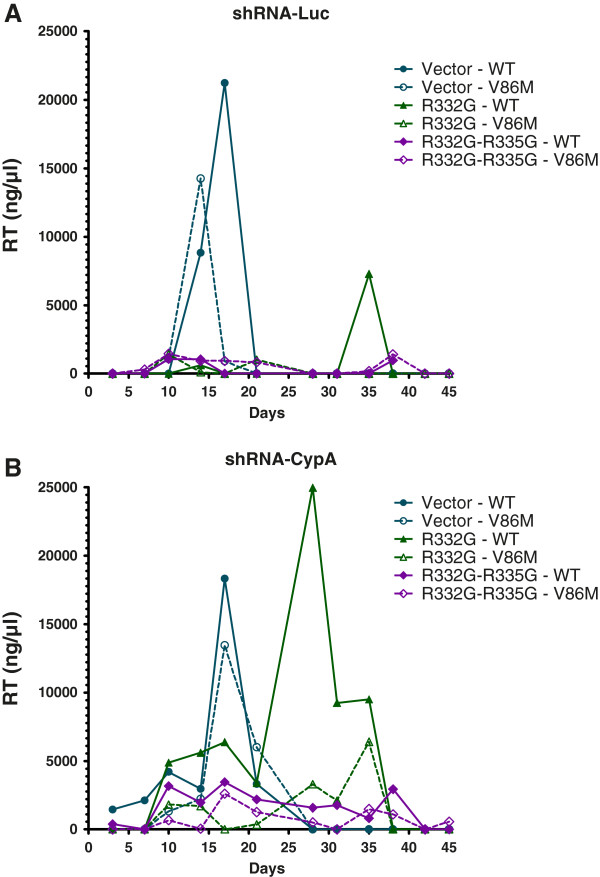
**Restriction of a spreading CA-V86M HIV-1 infection. **Sup-T1 cells transduced with a control shRNA against luciferase (**A**) or with an shRNA targeting CypA (**B**) and expressing the indicated TRIM5α proteins were infected once with an identical dose of WT or V86M HIV-1_NL4-3_ normalized by RT activity. Unbound virus was eliminated 16 h later and infection was allowed to proceed for 45 days. Reverse transcriptase activity in the supernatant of infected cells and of uninfected cells as a control were determined periodically. Background noise as determined from the uninfected cells was subtracted from the other samples at each time-point.

Single-cycle experiments using HIV-1 vectors are useful to investigate restriction events taking place between entry and integration. However, restriction of fully replicating HIV-1 may be more complex than what is reflected in these assays. Indeed, TRIM5α may also interfere with late stages of the retroviral cycle [46] although these conclusions have been rebutted [47]. Perhaps even more significantly, HIV-1 propagation occurs mainly through so-called virological synapses forming between cells [48-50]. It is still unclear whether TRIM5α restricts HIV-1 originating from these viral synapses in the same way that it targets incoming capsids from cell-free viruses, but there are some indications that it does not [51]. In order to investigate the role of CypA and the effect of V86M on HIV-1 restriction in a spreading infection context, we infected Sup-T1 cells with the NL4-3 strain of HIV-1 (Figure 8). The cell lines included in the experiment expressed R332G or R332G-R335G TRIM5α_hu_ and were co-transduced with the shRNA against CypA or the control shRNA targeting luciferase. The V86M mutation in CA prevented the use of a sensitive CAp24 assay to monitor replication. Instead, we performed a reverse transcriptase (RT) assay [38]. Replication of WT NL4-3 in the control (vector) shRNA-Luc cells peaked at about 18 days post-infection, while replication of the V86M mutant in the same cells peaked at about 15 days. Thus, V86M did not affect the fitness of HIV-1 in Sup-T1 cells. Replication of either WT or V86M NL4-3 was not detectable in the shRNA-Luc cells also expressing R332G-R335G TRIM5α_hu_ (Figure 8, top panel). Therefore, if the V86M mutation conferred some level of resistance to R332G-R335G TRIM5α_hu_ in a spreading infection context, it did not do so at levels high enough to allow detection of replication. In cells expressing R332G TRIM5α_hu_, active replication was detected but only at a single time-point (day 35 post-infection). Replication of V86M NL4-3 was not detected in the same cells, which suggests that V86M does not provide protection against this particular mutant of TRIM5α_hu_ in a spreading infection assay. In cells transduced with the shRNA targeting CypA, viral replication was delayed compared to the control shRNA-Luc cells, as expected. This was more obvious for the V86M virus which peaked at about 18 days, confirming that CA-V86M HIV-1 depends on CypA for optimal replication in human cells. Interestingly, replication of WT and V86M NL4-3 in shRNA-CypA/R332G TRIM5α_hu_-expressing cells was highly increased compared with replication in the shRNA-Luc cells. Specifically, replication of WT NL4-3 peaked at 28 days post-infection, compared to 35 days post-infection in the control cells, and the amount of viruses in the supernatants was also much higher. V86M NL4-3 peaked at about 35 days post-infection in the shRNA-CypA/R332G TRIM5α_hu_-expressing cells while its replication was not detected in the shRNA-Luc/R332G cells. This suggests that CypA knockdown can rescue replication of these two viruses in TRIM5-restrictive conditions and also that V86M NL4-3 is more sensitive to restriction by R332G TRIM5α_hu_, in contradiction with the data obtained in vector transduction experiments. In shRNA-CypA/R332G-R335G TRIM5α_hu_-expressing cells, replication of both WT and V86M NL4-3 was detected, although at low levels. Replication of V86M NL4-3 was no more efficient than that of its WT counterpart in these cells. Altogether, this experiment shows that restriction of a spreading HIV-1 infection by mutant TRIM5α_hu_ in Sup-T1 cells is enhanced by CypA, and suggests that the V86M mutation does not rescue HIV-1 replication in this context.

## Discussion

HIV-1 resistance to treatment is a hallmark of pharmacological interventions against this virus. Invariably, mutations appear in the coding sequences of the proteins targeted by antiretroviral drugs, including protease, reverse transcriptase and integrase [52]. It is expectable that genetic interventions will similarly lead to the occurrence of viral resistance. Indeed, restriction of HIV-1 by CPSF6-358, a truncated form of the RNA processing factor, cleavage and polyadenylation specific factor 6 (CPSF6), is counteracted by the mutation N74D in CA [53]. This mutation, isolated by serial passages of HIV-1 in cells expressing CPSF6-358, abrogates direct binding of CA to CPSF6-358 [54]. Several groups, including ours, have produced mutants of TRIM5α_hu_ to be used as antiviral transgenes [36,38-41]. In order to predict potential HIV-1 escape from inhibition mediated by these TRIM5α_hu_ variants, it is important to isolate those escape mutants *in vitro*, and to understand by which mechanism they decrease sensitivity to restriction. Our initial efforts had not led us to isolate R332G-R335G TRIM5α_hu_-resistant HIV-1 by serial passaging. We then decided to test whether V86M, an HIV-1 CA mutant isolated from cells expressing TRIM5α_rh_ by the Sodroski group and shown to confer some level of protection against this orthologue_,_ would also make HIV-1 resistant to TRIM5α_hu_ mutants.

Our data show that V86M indeed confers some level of protection against various mutants of TRIM5α_hu_, at least in the context of single-cycle infections with HIV-1 vectors. We saw no protection in the context of HIV-1 spreading infections, although we tested only one HIV-1 strain (NL4-3) in one cell line (Sup-T1). Sodroski and collaborators [35] have isolated the V86M mutant in HeLa-CD4 cells, which we have not tested here. They observed modest levels (2-fold) of protection against TRIM5α_rh_ in these HeLa-derived cells when infectivity was measured in single-cycle assays, and they saw an even more modest effect in canine Cf2Th cells [35]. Altogether, V86M can confer HIV-1 protection against restriction by various TRIM5α proteins in specific replication settings. However, even in situations in which protection takes place, restriction still occurs, albeit weakened. On the basis of our data, we do not expect V86M to be highly significant in an *in vivo* context, although this is of course rather hazardous to predict.

In order to investigate the mechanism of CA-V86M HIV-1 resistance to R332G-R335G TRIM5α_hu_, we analyzed the role of CypA in the restriction. Altogether, our results show that restriction of V86M CA HIV-1 is largely insensitive to the presence of functional CypA, while the same virus is still inhibited in human cells devoid of CypA. In other words, HIV-1 is able to subtly alter its interactions with CypA in order to downregulate a mechanism of restriction while preserving other benefits associated with this interaction. Accordingly, our NMR data showed that the molecular interactions between CA and CypA were altered by the V86M mutation, as was the isomerisation reaction catalysed by CypA.

Finally, our qPCR data correlate modifications in CypA-CA interactions with effects on nuclear transport in restrictive conditions. Interestingly, experiments pre-dating the discovery of TRIM5α restriction had demonstrated that CsA treatment of Old World monkey cells increased nuclear transport of HIV-1 in these cells [21]. More recently, Lin and Emerman similarly observed that CsA treatment of the sMAGI Rhesus macaque cell line increases HIV-1 nuclear transport more than it does enhance reverse transcription [55]. Other recently published data also link nuclear transport and CA-CypA interactions in permissive human cells. Specifically, CypA knockdown and CsA treatment reduce HIV-1 dependency toward nucleopore components Nup153 and Nup358 for its nuclear transport [56,57]. It should be informative to analyze whether V86M modifies the interactions between HIV-1 CA and these nucleoporins.

## Conclusions

The V86M mutation in HIV-1 CA can confer partial resistance against restriction of HIV-1 replication by mutants of human TRIM5α. V86M abrogates CypA-dependent restriction mechanisms, resulting in an increase of HIV-1 DNA nuclear transport in restrictive conditions. However, this mutation might not confer significant resistance to the restriction of a spreading HIV-1 infection.

## Methods

### Plasmid DNAs

pMIP-TRIM5α_hu_ and pMIP-TRIM5α_rh_ express C-terminal FLAG-tagged versions of the corresponding proteins and have been extensively described before [28,38,44,58,59]. pMIP-TRIM5α_hu_ with the mutation R332G-R335G has been described [38], and additional mutants have been described as well [42]. pSRBl-CypA expresses a short hairpin RNA (shRNA) targeting the human CypA mRNA. Shortly, it is the previously described pSRP-CypA plasmid [27] in which the puromycin resistance cassette has been changed to one conferring resistance to blasticidin. The control plasmid, pSRBl-Luc, encodes a shRNA targeting luciferase [60]. pNL-GFP, pMD-G and pCL-Eco have all been described elsewhere [21,28,58,61-63]. The fully replication-competent HIV-1 clone pNL4-3 [64] was used for propagation experiments.

### Cells

Human rhabdomyosarcoma TE671 cells, human embryonic kidney 293 T cells and feline renal CRFK cells were maintained in Dulbecco’s modified Eagle’s medium supplemented with 10% fetal bovine serum and antibiotics at 37°C. Human T lymphoblast Sup-T1 cells and monocytic cells THP-1 were maintained in RPMI-1640 supplemented with 10% fetal bovine serum and antibiotics at 37°C. All cell culture reagents were from Hyclone (Thermo Scientific, Logan, UT, USA). Human PBMCs were isolated from diluted whole blood of a healthy donor by Ficoll density gradient centrifugation as previously described [65]. CD4^+^ T cells were then isolated using a negative isolation kit (Dynabeads Untouched Human CD4 T Cells, Invitrogen) according to manufacturer specifications. The isolated cells were cultivated in RPMI-1640 supplemented with 10% fetal bovine serum and antibiotics. Activation and proliferation of CD4^+^ T cells were performed by supplementing the culture medium with 60 U/ml IL-2 (Peprotech, Canada) and incubating the cells with anti-CD3/CD28 coated beads (Dynabeads Human T-Activator CD3/CD28, Invitrogen) according to the manufacturer instructions. Infections of these cells were performed in the absence of IL-2 and activating beads. THP-1 monocytic cells (7.5 x 10^5^) were differentiated into M0 macrophages by incubation in serum-free medium supplemented with 50 ng/ml PMA and 10 ng/ml GM-CSF for 48 h. Polarization into pro-inflammatory M1 macrophages was induced by addition of 100 ng/ml LPS and 25 U/ml IFN-γ. Anti-inflammatory M2 macrophages were induced by addition of 20 ng/ml IL-4 and 20 ng/ml IL-10 (all reagents were from Peprotech). In all cases, non-adherent cells were washed away before infection.

### Retroviral vectors production

HIV-1 and MLV-based vectors were produced through transient transfection of 293 T cells and collected as previously described [38,44]. To produce HIV-1_NL-GFP_, cells were co-transfected with pNL-GFP and pMD-G. To produce the MLV-based vectors SRBl and MIP, cells were transfected with the relevant pMIP or pSRBl plasmid and co-transfected with pCL-Eco and pMD-G. To produce HIV-1 VLPs, cells were co-transfected with pΔR8.9 and pMD-G. When necessary, amounts of viruses were normalized using the nonradioactive EnzChek RT Assay Kit (Invitrogen, Burlington, ON), or based on their titer in permissive human cells.

### RNA interference

pSRBl-based retroviral vectors encoding an shRNA targeting either CypA or Luc mRNAs were produced in 293 T cells as described [28,38]. TE671 and Sup-T1 cells were plated at 250,000 cells per well in 12-well plates and exposed to 1 mL of either SRBL-CypA or -Luc vector preparation. Two days post-transduction, cells were placed in medium containing 10 μg/ml of blasticidin (Sigma-Aldrich). Selection was allowed to proceed for 1 week. Efficiency of the knockdown was verified by assaying CypA expression levels in the transduced cells by western blotting, using antibodies directed against CypA (rabit polyclonal IgG; Sigma-Aldrich, StLouis, MI) and actin (mouse monoclonal; Chemicon International).

### Viral challenges

Cells were plated in 24-well plates at 50,000 cells/well (TE671 and CRFK), 100,000 cells/well (Sup-T1), 200,000 cells/well (primary CD4 T cells) or 750,000 cells/well (differentiated THP-1) and infected with HIV-1 vectors (either HIV-1_TRIP-CMV-GFP_ or HIV-1_NL-GFP_) with or without CsA treatment (2 or 5 μM). Drugs were added 15 min prior to infections. In CsA dose-dependent experiments, virus doses were adjusted for each virus-cell combination so that approximately 1% of the cells would be infected in the absence of CsA. Two days post-infection, cells were trypsinised when necessary, fixed in 1 to 2% formaldehyde in a PBS solution. The % of GFP-positive cells were then determined by analyzing 10,000 to 20,000 cells with a FC500 MPL cytometer (Beckman Coulter) using the CXP software (Beckman Coulter). For spreading infections, quantification of RT activity in the supernatants of infected cells was done exactly as before [38].

### Quantitative real-time PCR of HIV-1 DNA

Cells were plated in 12-well plates at 3x10^5^ cells/well and infected with HIV-1_NL-GFP_ WT or V86M vectors. Viruses were passed through 0.45 μm filters and pretreated for 1 hour at 37°C with 20U/ml DNAse I (New England Biolabs) to prevent contamination by carry-over plasmid DNA. In addition, control infections were performed in presence of 80 μM nevirapine to ascertain the absence of such contaminating plasmid DNA. Total cellular DNA was prepared after 6 hours of infection (late RT products) or 6 hours of infection followed by 18 hours of incubation in virus-free medium (2LTR-circles) and DNA preparation was done using the DNeasy Blood and Tissue Kit (Qiagen, California).

The primer sets to detect each DNA species were as follows: GFP forward, GACGACGGCAACTACAAGAC; GFP reverse, TCGTCCATGCCGAGAGTGAT; 2LTR-circles forward (MH535), AACTAGGGAACCCACTGCTTAAG [66]; 2LTR-circles reverse (MH536), TCCACAGATCAAGGATATCTTGTC; GAPDH forward, GTCAGTGGTGGACCTGACCT; GAPDH reverse, TGAGCTTGACAAAGTGGTCG. In each experiment, a standard curve of the amplicon being measured was run in duplicate ranging from 30 to 3 × 10^5^ copies plus a no-template control. Reactions contained 1× SensiFAST SYBR Lo-Rox kit (Bioline, UK), 300nM forward and reverse primers, and 5 μl template DNA (100-300 ng) in 20 μl-sized reactions. After initial incubation step of 3 min at 95°C, 40 cycles of amplification were carried out at 10s at 95°C, 10s at 62°C (GFP) or 65°C (2LTR, GAPDH) and 10s (GFP) or 15 s (2LTR, GAPDH) at 72°C on a MX3000P qPCR system (Agilent Technologies, California). Results were analyzed with the MxPro software (Agilent Technologies).

### Statistical analyses

Statistical data were calculated using GraphPad Prism version 5.03. Student’s unpaired t-tests were used for tests of difference between means.

### 2D ^1^H-^15^ N Heteronuclear (ZZ) Exchange Spectroscopy

Uniformly ^15^ N-labeled CA^N^ domain of HIV1 (^15^ N HIV1caN) was expressed in DE3 bacteria in K-MOPS buffer supplemented with 20 mM ^15^NH_4_Cl as a sole source of nitrogen, 4 mM pH 8.0 KPOi, 0.1 ^o^/_oo_ sodium ampicillin salt, vitamins and 0.4% glucose. Bacterial culture was grown until 0.500-0.700 OD_600_ at 37°C, induced with 1 mM Isopropyl β-D-1-thiogalactopyranoside (IPTG), and the protein was expressed at 20°C overnight followed by purification as previously described [67] except that the final step of purification, i.e. gel filtration, was run in 50 mM potassium phosphate, pH 6.5, 100 mM NaCl and 1 mM DTT. Uniformly ^15^ N-labeled CA^N^ domain of HIV1 V86M (^15^ N HIV1caN V86M) was expressed and purified as per ^15^ N HIV1caN. Non-isotopically labeled N-terminally his_6_-tagged human CypA was expressed in DE3 bacteria in 2xTY medium supplemented with 0.6% glucose and 0.1 ^o^/_oo_ sodium ampicillin salt, induced at 0.6-0.8 OD_600_ at 37°C with 1 mM IPTG, expressed at 20°C and purified using Ni-NTA beads (Qiagen) and gel-filtration chromatography in 75 mM Tris pH8.0, 50 mM NaCl and 1 mM DTT buffer. For all NMR experiments, proteins were dialysed against the same 50 mM KPOi, pH 6.5 buffer and supplemented with 1 mM DTT. The capsids were used at 12-fold excess concentration over CypA as previously described [33,68]: 430 μM CA^N^/35 μM Cyp A or 430 μM CA^N^ only. All NMR samples contained 5% D_2_O.

2D ^1^H-^15^N heteronuclear (ZZ) exchange experiment is described in detail in [69] and was previously applied for a similar model [33,68]. The experiments were performed on a Bruker 800 MHz spectrometer at 298 K using in-house written pulse program with the following mixing times in a randomised order: 3 ms, 25 ms, 47 ms, 69 ms, 75 ms, 97 ms, 147 ms, 169 ms, 197 ms, 247 ms, 297 ms, 397 ms, 547 ms and 796 ms. The first time-point was acquired twice to assess the error. G89 assignment for ^15^ N HIV1caN was assumed from ppm chemical shifts referred to in [33,68]. It was also assumed that chemical shifts for G89 in ^15^ N HIV1caN V86M did not change dramatically. The data was processed in TopSpin 2.0 (Bruker, Karlsruhe) and after analysis in Sparky (T. D. Goddard and D. G. Kneller, University of California, San Francisco).

## Competing interests

The authors declare that they have no competing interests.

## Authors’ contributions

LB, LCJ and MV designed the experiments, interpreted the results and wrote the manuscript. MV, KB, PP, SMVF, MBP, QTP, CRM and LB performed the experiments. All authors read and approved the final manuscript.

## Supplementary Material

Additional file 1: Figure S1 shows knockdown of CypA in TE671 and in Sup-T1 cells. **Figure S2** shows the effects of V86M and of CsA on the restriction of HIV-1 by additional mutants of TRIM5α_hu_. Click here for file
