# Supplementary material for: The V86M mutation in HIV-1 capsid confers resistance to TRIM5α by abrogation of cyclophilin A-dependent restriction and enhancement of viral nuclear import
Source: Retrovirology. 2013 Feb 28;10:25. doi: 10.1186/1742-4690-10-25 (PMC3598646; doi:10.1186/1742-4690-10-25)
Supplement: Additional file 1: Figure S1 — shows knockdown of CypA in TE671 and in Sup-T1 cells. Figure S2 shows the effects of V86M and of CsA on the restriction of HIV-1 by additional mutants of TRIM5αhu. [file 1742-4690-10-25-S1.pdf]

Veillette et al.

The V86M mutation in HIV-1 Capsid Confers Resistance to TRIM5α by Abrogation of Cyclophilin A-Dependent Restriction and Enhancement of Viral Nuclear Import.

Supplementary data

Figure S1. CA-V86M HIV-1 is inhibited by CsA in human lymphocytes and macrophages. (**A**) Activated human $CD4^+$ lymphocytes were challenged with WT or V86M HIV-$1_{NL\text{-}GFP}$ at a multiplicity of infection of ~0.1 in the absence or presence of 2 µM CsA. WT and V86M viral preparations were adjusted by reverse transcriptase assay. The percentage of infected (GFP-positive) cells was determined two days later by flow cytometry. Shown are average values from three independent infections with standard deviations. (**B**) THP-1 cells differentiated and polarized into either macrophages (M0), pro-inflammatory macrophages (M1) or anti-inflammatory macrophages (M2) were challenged with WT or V86M HIV-$1_{NL\text{-}GFP}$ exactly like above but at an M.O.I of ~0.02.

Figure S2. Western blotting analysis of CypA knockdown. TE671 cells (top) and Sup-T1 cells (bottom) expressing the indicated TRIM5α_{hu} mutants and controls were stably transduced with retroviral vectors expressing shRNAs targeting either CypA or the non-relevant control Luciferase. Untransduced cells were eliminated; then, whole cell lysates were prepared from a similar numbers of cells and processed for western blotting using antibodies against CypA or X-actin as a loading control.

Figure S3. Restriction of WT and CA-V86M HIV-1 by a panel of TRIM5α_{hu} mutants. (**A**) TE671 cells transduced with the indicated TRIM5α cDNAs were challenged with multiple doses of WT or CA-V86M HIV-$1_{NL\text{-}GFP}$ as described in Figure

1. Cells were analyzed by FACS 2 days later. Control permissive cells transduced with the “empty” vector and infected with WT HIV-1_{NL-GFP} are included. (**B**) TE671 cells expressing the indicated TRIM5α_{hu} mutants and controls were challenged with WT or V86M HIV-1_{NL-GFP} in the presence of increasing concentrations of CsA. Virus doses were adjusted for each virus-cell combination so that approximately 1% of the cells were infected in the absence of CsA. The percentage of GFP-positive cells was determined by FACS 2 days later and results are shown as –fold increases relative to the no-drug controls.

S1

A

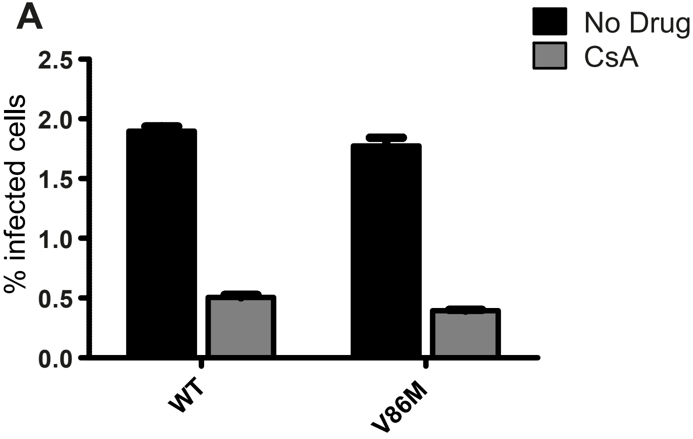

No Drug
CsA
% infected cells
2.5
2.0
1.5
1.0
0.5
0.0
WT
V86M

B

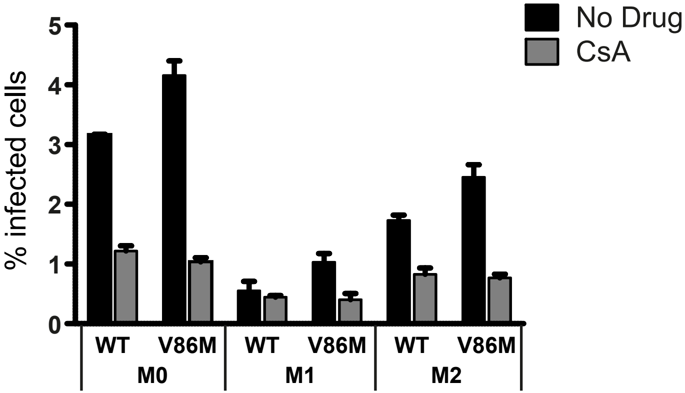

No Drug
CsA
% infected cells
5
4
3
2
1
0
WT
V86M
WT
V86M
WT
V86M
M0
M1
M2

S2

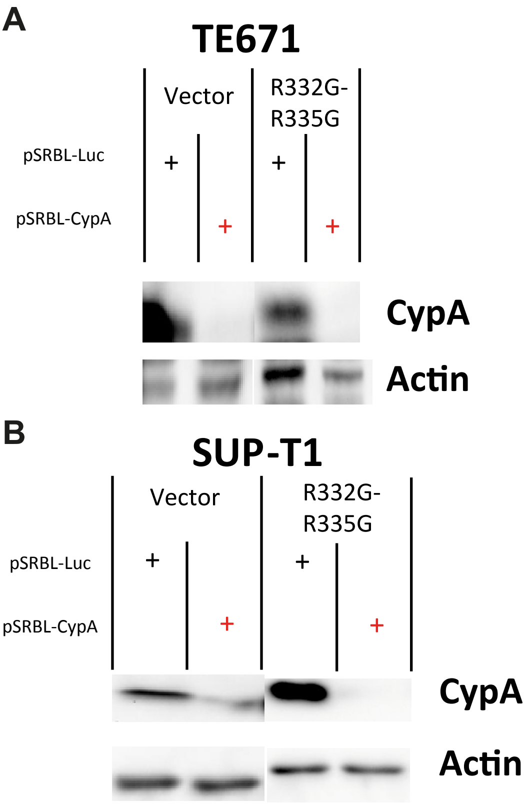
A
TE671
Vector
R332G-
R335G
pSRBL-Luc
+
+
pSRBL-CypA
+
+
CypA
Actin
B
SUP-T1
Vector
R332G-
R335G
pSRBL-Luc
+
+
pSRBL-CypA
+
+
CypA
Actin

S3

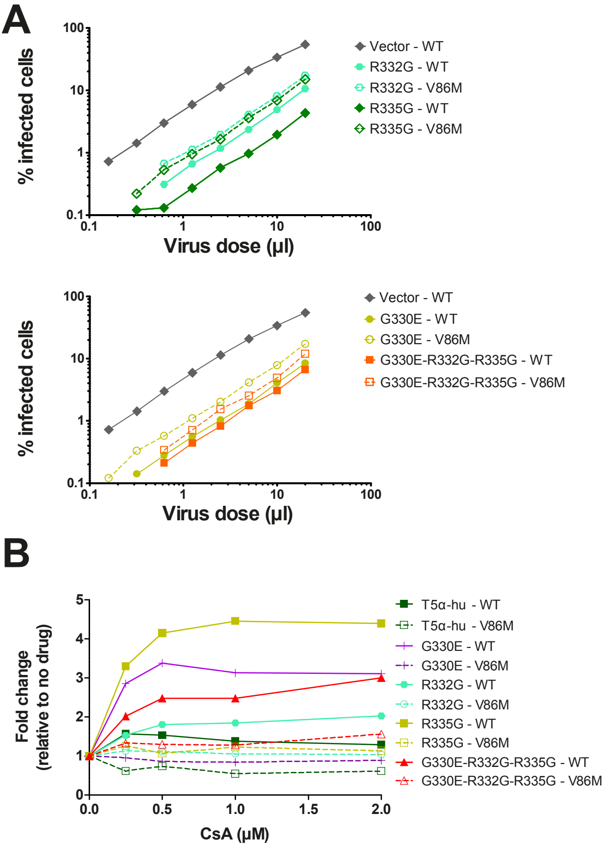
A
100
10
1
0.1
0.1
1
10
100
% infected cells
Virus dose (µl)
Vector - WT
R332G - WT
R332G - V86M
R335G - WT
R335G - V86M
100
10
1
0.1
0.1
1
10
100
% infected cells
Virus dose (µl)
Vector - WT
G330E - WT
G330E - V86M
G330E-R332G-R335G - WT
G330E-R332G-R335G - V86M
B
5
4
3
2
1
0
0.0
0.5
1.0
1.5
2.0
Fold change
(relative to no drug)
CsA (µM)
T5α-hu - WT
T5α-hu - V86M
G330E - WT
G330E - V86M
R332G - WT
R332G - V86M
R335G - WT
R335G - V86M
G330E-R332G-R335G - WT
G330E-R332G-R335G - V86M
